# The Fabrication of Micro-Array Channels with the Ultrafine-Grained LZ91 Mg-Li Alloy by Micro-Embossing

**DOI:** 10.3390/mi9020055

**Published:** 2018-01-31

**Authors:** Qian Su, Jie Xu, Chenxi Wang, Debin Shan, Bin Guo

**Affiliations:** 1 Key Laboratory of Micro-Systems and Micro-Structures Manufacturing of Ministry of Education, Harbin Institute of Technology, Harbin 150080, China; susan8401@163.com (Q.S.); beyondchenxi@163.com (C.W.); shandebin@hit.edu.cn (D.S.); guobin@hit.edu.cn (B.G.); 2 School of Materials Science and Engineering, Harbin Institute of Technology, Harbin 150001, China

**Keywords:** micro-embossing, ultrafine grains, micro-array channels, Mg-Li alloy

## Abstract

The fabrication of the micro-channel array through micro-embossing on an ultrafine-grained (UFG) LZ91 Mg-Li alloy was investigated in this paper. Micro-embossing tests indicated that the depth of the channels increase with increasing temperatures. Micro-array channels with widths ranging from 50 μm to 200 μm were performed with an applied force of 2 kN at 423 K, following by a dwell time of 60 s on the UFG LZ91 Mg-Li alloy. The surface topography indicates that embossed micro-channels for UFG LZ91 Mg-Li with all widths are clearly printed well with good geometrical transferability and no obvious defects. The comparison filling behavior of UFG and the CG LZ91 Mg-Li alloy reveals that grain sizes mainly affect the filling quality of micro-embossing. The results indicate a potential application of the UFG Mg-Li alloy in the mass fabrication of micro-electro-mechanical system (MEMS) components.

## 1. Introduction

The micro-channels array has the potential to be used in the matter of micro-fluidics such as components of micro-reactors or micro heat exchangers; or in micro-optics such as optical grating, which is an important component of micro-electro-mechanical systems (MEMS) [1,2,3]. To enhance the heat transfer efficiency, some metals with a high conductivity and low cost, like magnesium, have been widely used in the fabrication of micro-array channels [4]. For magnesium alloys, they have good properties such as low density, high damping capacity, high cycling capacity, and lower energy requirements [5,6]. However, due to their hexagonal close-packed (HCP) structure and the operation of limited slip systems, the formability of magnesium alloys at an ambient temperature is poor [7]. Hence, poor ductility and poor cold workability at an ambient temperature restricts their wider application [8,9]. The addition of lithium (Li) to Mg alloys reduces the c/a axial ratio of the HCP lattice and introduces phases with a body-centered cubic crystal structure with more slip systems, which can effectively improve the deformation performance [10]. On the other hand, the addition of Li will reduce the strength of magnesium alloys. Indeed, Mg-Li alloys are the lightest structural materials with a high stiffness ratio, good machining property, excellent magnetic screening, and ability to resist shock. Mg-Li alloys are attracting more and more interest in terms of both scientific research and industrial applications such as the aerospace, military, and electronic industries [11].

Micro-embossing is a promising process for obtaining functional surface microstructures due to its capacity actualize low-cost, highly-efficient, large-scale, and well-structured fabrications [12]. Neugebauer et al. [13] developed a micro-embossing technique using a silicon die to fabricate metallic microstructures. Otto et al. [14] investigated the fabrication of a micro-optical grating by a high-precision cold embossing method in pure coarse-grained (CG) Al using silicon die, in which the fabricated channels exhibited non-flat planks with ridges. Böhm et al. [15] further studied the fabrication of straight micro-channels and micro-complex structures by cold and superplastic micro-embossing with silicon die. Jiang et al. [16] researched the fabrication of micro-array channels by direct micro-embossing in conventional CG Al at an ambient temperature. Qiao et al. [17] studied the micro-embossing process on an ultrafine-grained (UFG) aluminum alloy and fabricated micro-array channels for MEMS components. Xu et al. [4] investigated the hot micro-embossing process of micro-array channels on UFG pure Al with a balance device. Wang et al. [18] researched the influence of die cavity size on formability during the warm coining process, which indicates that the formability decreases with the increasing ratio of grain size to die cavity width. Wang et al. [19] investigate the size effects of micro-bulk formation by proposing a multi-region model that considered the grain orientation and boundary by micro-coining process. Gao et al. [20] investigated the influence of grain and geometry size effects on deformation during roll-to-plate micro/meso-imprinting of pure copper. From previous research, it can be seen that the grain size is the main factor determining the limit size of the geometric characteristics of the micro forming.

Nevertheless, research about using micro-embossing to fabricate micro-channels array on the UFG Mg-Li alloy is limited. In this work, micro-array channels with widths ranging from 50 μm to 200 μm were fabricated by micro-embossing process on the UFG Mg-Li alloy processed by HPT using a metal die. The experimental results indicate that there is potential for using the UFG Mg-Li alloy in the mass production of micro-array channels for MEMS components.

## 2. Materials and Methods

The experiments were conducted using a commercial LZ91 Mg-Li alloy supplied in the form of drawn rods with a diameter of 10.0 mm. HPT processing was adopted to achieve UFG LZ91 Mg-Li alloy using an imposed pressure of 6.0 GPa through 10 turns at ambient temperature at a rotation rate of 1 rpm. After HPT processing, the micro-embossing samples were machined by electric discharge machining (EDM) to produce small blocks with dimensions of 5.5 (length) mm × 3.5 (width) mm. Before the micro-embossing tests, the specimens were mounted, ground on SiC papers, and then mechanically polished. Lastly, the specimens were electropolished to mirror-like surfaces with a solution of H_3_PO_4_ and C_2_H_5_OH (volume ratio was 3:5) under a DC voltage of 5 V at ambient temperature.

The microstructure of the original Mg-Li alloy was observed by metallographic microscope (OLYMPUS, GX71, Olympus Company, Tokyo, Japan). Transmission electron microscopy (TEM, Tecnai G2 F30, FEI Company, Hillsboro, OR, USA) was used to characterize the microstructure of the materials after HPT processing after 10 turns. Thin sections were taken from a position 2.5 mm from the center of the LZ91 Mg-Li alloy specimens after HPT processing followed by ion milling.

**Figure 1:**
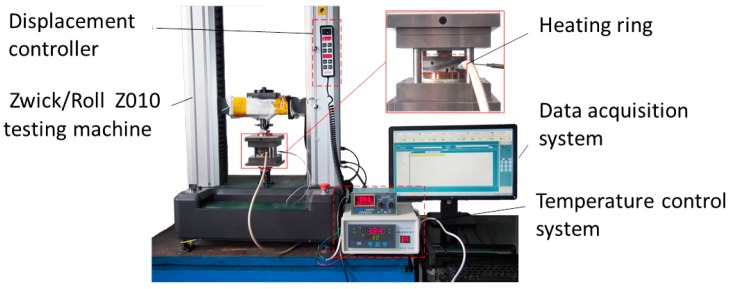
Testing system of micro-embossing.

**Figure 2:**
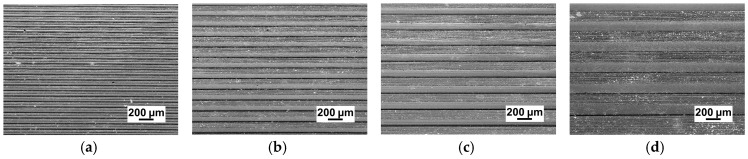
SEM of micro-array channels dies with different widths: (**a**) 50 μm; (**b**) 100 μm; (**c**) 150 μm; (**d**) 200 μm.

**Figure 3:**
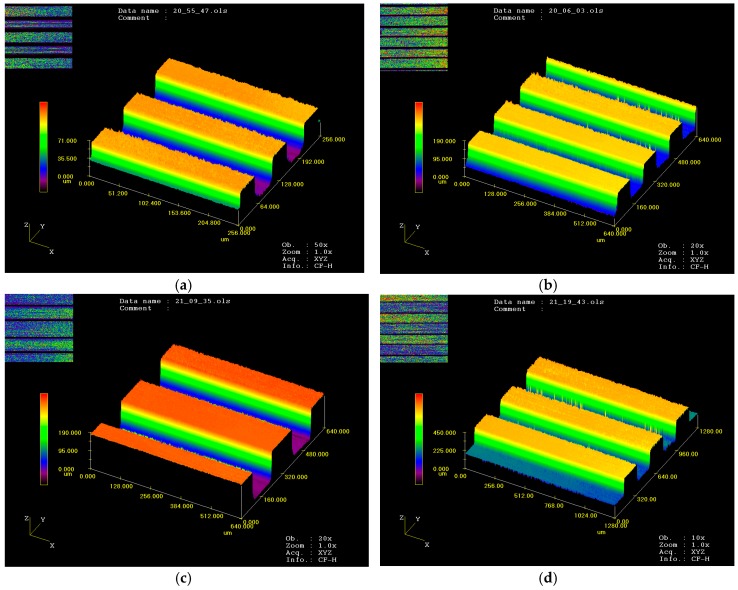
Profile measurements of micro-array channels dies with different widths: (**a**) 50 μm; (**b**) 100 μm; (**c**) 150 μm; (**d**) 200 μm.

The micro-embossing tests were performed at ambient temperature on a Zwick/Roll Z010 testing machine, as shown in Figure 1. The micro-embossing tools used in this paper were metal dies that were fabricated by machining. The micro metal dies are a sequence of parallel straight channels with sizes of 50 μm × 50 μm, 100 μm × 100 μm, 150 μm × 150 μm, and 200 μm × 200 μm, referring to width and depth, respectively. The space between two adjacent channels is equal to the width of channels for all channels based on the previous research [4]. The surface morphology of the micro metal dies was examined by an FEI Quanta 200FEG field emission SEM and a laser confocal scanning microscope. The SEM and 3D profile photographs show that all micro-array channels with four different widths have excellent surface quality and high profile precision, as shown in Figure 2 and Figure 3. From the results, it can be seen that the micro metal die has good surface quality without obvious defects, and side walls of channels are exactly 90° with regular outline. These results indicate these micro metal dies can meet the requirements of the micro-embossing process without the influence of die defects.

**Figure 4:**
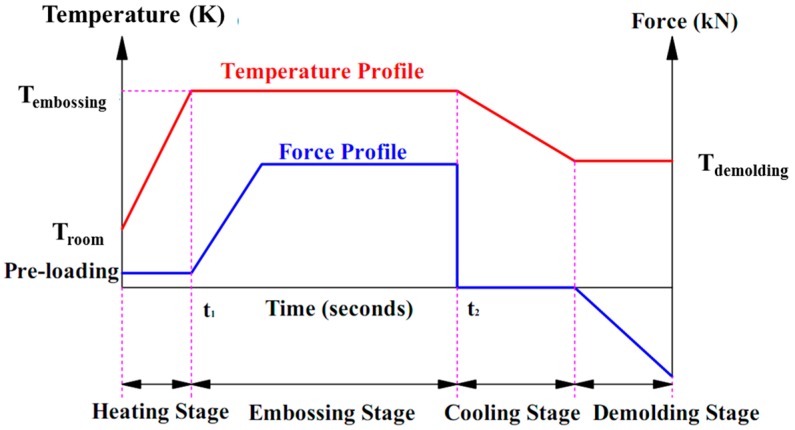
Process curve of micro embossing.

To obtain the optimum micro-embossing parameter of UFG LZ91 Mg-Li alloy, the applied force was 2.0 kN at elevated embossing temperatures of 373, 398, and 423 K with a hold time of 60 s, which the process curve was shown in Figure 4. During loading and holding process, the temperature was kept on the embossed temperature. After micro embossing process, the surface quality of embossed specimens with micro-channels was observed by an FEI Quanta 200FEG field emission SEM and a laser confocal microscope (OLS3000, Olympus Company, Tokyo, Japan).

## 3. Results

### 3.1. Microstructure of UFG LZ91 Mg-Li Alloy

**Figure 5:**
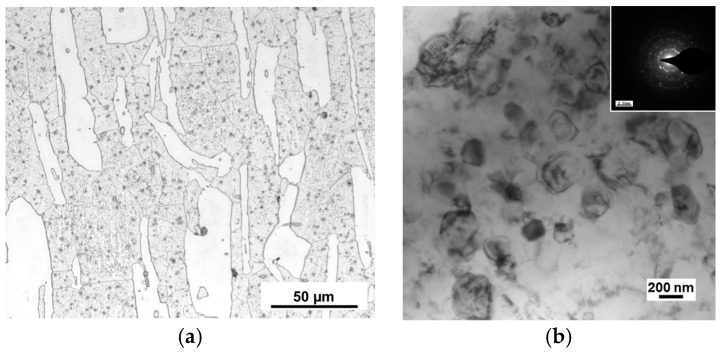
Microstructure of Mg-Li alloy: (**a**) OM images original condition; (**b**) TEM observation processed by HPT after 10 turns.

The microstructure of the original Mg-Li alloy is shown in the optical micrographs (OM) in Figure 5a, in which the white regions are α phase and the gray regions are β phase, and long strip α phase is distributed on β phase. Based on the result, volume ratio of α and β phase is 28% and 72%, respectively. Meanwhile, the mean linear intercept grain size was approximately 22 μm.

The microstructure of LZ91 Mg-Li alloy processed by HPT after 10 turns was studied using TEM, and the result is shown in Figure 5b. Significant grain refinement is observed after HPT processing through 10 turns, with an average grain size of approximately 250 nm, as shown in Figure 5b. Meanwhile, the microstructure of Mg-Li alloy after 10 turns of HPT processing becomes homogeneous, and the selected area electron diffraction (SAED) patterns in the illustration of Figure 5b indicate that the grain boundaries have high angles of misorientation. Result at present is in line with earlier research that described that an Mg-8%Li alloy with a grain size of ~500 nm after HPT through 5 turns at ambient temperature [21] and an AZ80 alloy with an average grain sizes of ~200 nm after HPT through ten turns at ambient temperature [22].

### 3.2. Micro-Embossing Process on UFG LZ91 Mg-Li Alloy

**Figure 6:**
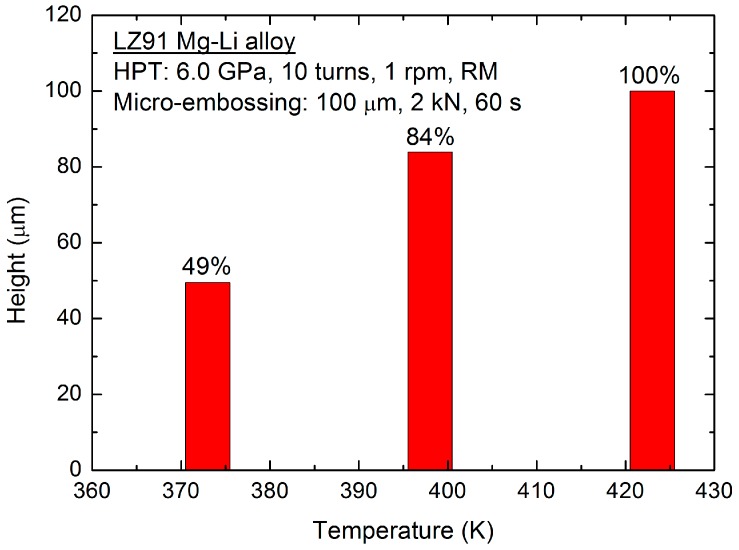
Filling height after micro-embossing with an increasing applied temperature.

Figure 6 shows the filling height of the embossed micro-channels on the UFG LZ91 Mg-Li alloy surface using micro metal dies under an applied force of 2 kN at different elevated temperatures. The results show that the height of the embossed micro-channels increases with increasing micro-embossing temperature. The height increases from ~49.5 μm to ~100 μm when the embossing temperature increases from 373 K to 423 K with a hold time of 60 s. With the increasing of temperature, the flow stress decreases and plasticity increases of LZ91 Mg-Li alloy, respectively. Therefore, the filling height increases with increasing temperature under the same applied force. When the embossing temperature increases to 423 K, the filling ratio of embossed channels can achieve 100% with excellent filling quality.

**Figure 7:**
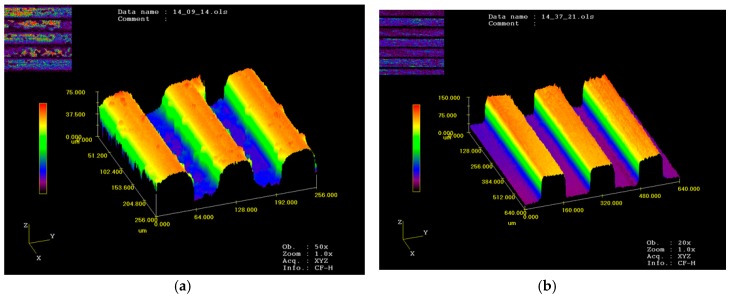

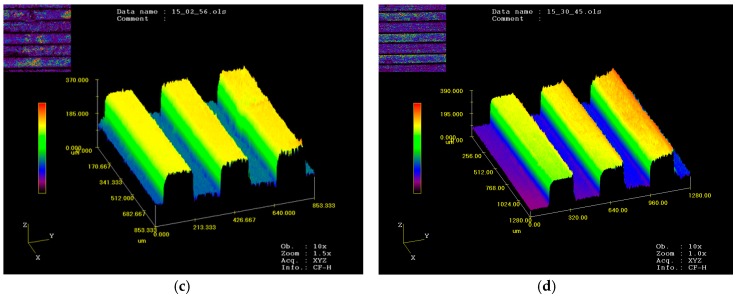
Profile measurements of micro-array channels with different widths: (**a**) 50 μm; (**b**) 100 μm; (**c**) 150 μm; (**d**) 200 μm.

Hence, the processes parameters of micro-embossing with different widths of channels for UFG LZ91 Mg-Li alloy are under an applied force of 2 kN at a temperature of 423 K, followed by a hold time of 60 s. The experimental results are shown in Figure 7 and Table 1. The results indicate that complete filling was observed for the embossed specimens with four different widths. Additionally, results of Figure 7 also indicate that the surface roughness for the channels becomes better with the increasing width of micro-channels. Meanwhile, in comparison of Figure 7 and Figure 3, it can be seen that the outline of channels is well transferred from the dies to the specimens, and the size accuracy is also very good. In addition, the channels on the metal die were well filled by the material of specimens, and results show that the dies and specimens can close together well.

### 3.3. Surface Topography of the Embossed Micro-Channels

**Figure 8:**
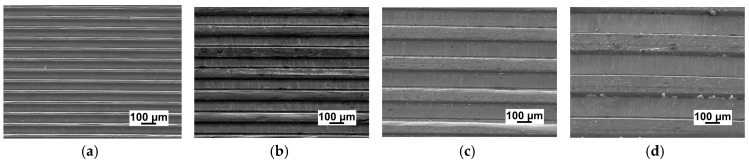
SEM images of micro-array channels with different width: (**a**) 50 μm; (**b**) 100 μm; (**c**) 150 μm; (**d**) 200 μm.

Figure 8 reveals the surface topography of embossed micro-channels on the UFG LZ91 Mg-Li alloy with channel width ranging from 50 μm to 200 μm by micro metal dies under a temperature of 423 K with an applied force of 2 kN, followed by a hold time of 60 s. The top surface of channels becomes a little rough for narrow channels, as shown in Figure 8a, which is in line with Figure 7a. With the increasing of channel width, the surface quality of embossed channels becomes better, showing good geometrical transferability from dies to samples, as shown in Figure 8b–d. Meanwhile, in comparison with Figure 2, the embossed channels are well transferred from the metal dies with high accuracy. In a word, the embossed micro-channels on the UFG LZ91 alloy surfaces with different widths are all embossed well with excellent transferability from dies to specimens and no obvious defects.

**Figure 9:**
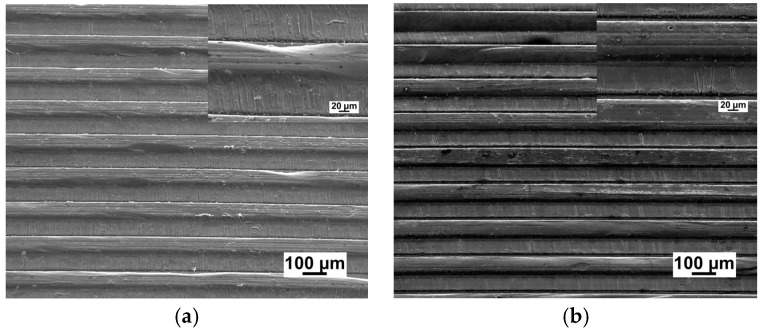
The comparison images of micro-channels on different materials: (**a**) CG LZ91 Mg-Li alloy; (**b**) UFG LZ91 Mg-Li alloy.

Figure 9 shows the SEM images of embossed channels under the same experimental conditions on CG and UFG LZ91 Mg-Li alloy. From the comparison of CG and UFG LZ91 Mg-Li alloy, obvious differences in the microstructure of the micro-channels are observed in Figure 9. Observation of Figure 9a indicates that the outlines of embossed channel on CG LZ91 Mg-Li alloy are uneven and there are some wrinkles on some channels. These results show that CG LZ91 Mg-Li alloy has poor formability during micro-embossing process, whereas the embossed channel on UFG LZ91 Mg-Li alloy is uniform without obvious defeats. The comparisons reveal that the UFG LZ91 Mg-Li alloy has much better formability than the CG LZ91 Mg-Li alloy.

## 4. Discussions

### 4.1. Formability of Micro-Embossing at Elevated Temperatures for UFG LZ91 Mg-Li Alloy

Significant grain refinement was achieved in LZ91 Mg-Li alloy with an initial grain size of ~22 μm. UFG LZ91 Mg-Li alloy with an average grain size of 250 nm was obtained after HPT processing under 6.0 GPa through ten turns at room temperature. One main purpose of this study was to evaluate the formability of UFG LZ91 Mg-Li alloy during micro-embossing process at elevated temperatures and then to identify that this material has potential applications in micro-forming. From the results shown in Table 1, ~100% filling was achieved for four different embossed micro-channels at a temperature of 423 K. Meanwhile, the patterns were fully transferred from micro metal die to the LZ91 Mg-Li alloy specimens for all widths of channels.

**Figure 10:**
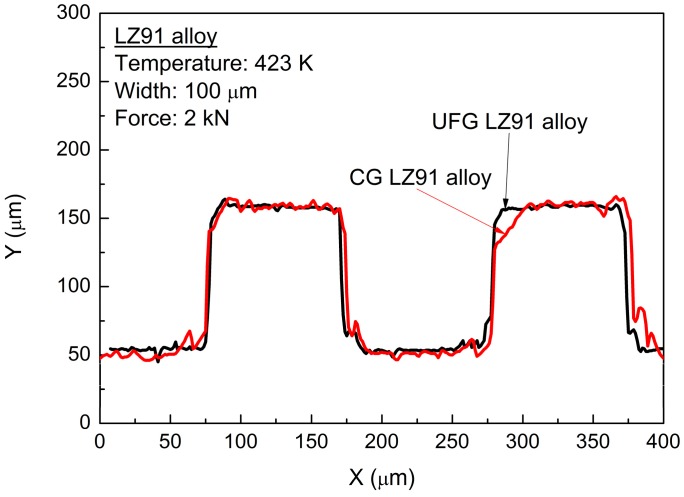
Comparison of the filling quality using CG and UFG Mg-Li alloy.

The materials flow in the transverse direction was restricted by the micro-metal mold, and the deformation of channels in vertical direction was free. With the increasing width of channels, the deformation mechanism was changed from shear deformation inside the grain to grain sliding and rotation in polycrystalline aggregates. Meanwhile, because of the increasing channel width, the flow behavior was improved obviously, so the fully transferred channels with excellent surface quality were obtained on UFG LZ91 Mg-Li alloy for four widths channels, as shown in Figure 6. Hence, much smaller channels should be fabricated by UFG materials with a much smaller grain size at the sub-micron scale. By comparison, the filling problem of embossed 50 μm channels in width is much more serious for CG LZ91 Mg-Li alloy in which there are some wrinkles and uneven channels, as shown in Figure 9a. For CG LZ91 Mg-Li alloy, similar dimension of grain size and width makes that there are several grains deformed during the micro-embossing, and this leads to nonuniform deformation in the interior of grains and poor compatibility of deformation between grains. Compared with CG specimens, the grain size of UFG specimens is only about 250 nm, and this ensures that there are a large number of grains deformed during the micro-embossing. Compatibility of deformation between grains is improved greatly, so that the wrinkles disappear. The profile measurements also indicate a similar result, as shown in Figure 10. The micro-channel profile for the CG LZ91 Mg-Li alloy is uneven and slanted in the transverse direction (red line), and the micro-channels profiles for UFG LZ91 Mg-Li alloy are straight and uniform (black line) as shown in in Figure 10. These results demonstrate that UFG LZ91 Mg-Li alloy has advantageous formability in the applications of micro-forming. 

### 4.2. The Potential Applications of UFG Mg-Li Alloy in Fabrication of MEMS Components

The other purpose of this research was to prove the potential applications of UFG LZ91 Mg-Li alloy in the mass fabrication of MEMS components by way of micro-embossing. The present results of research have revealed that embossed micro channels on UFG LZ91 Mg-Li alloy produced by HPT are sharp and smooth with good filling quality. The current process can be useful for implementation of mass production at low-cost by using the system without the expensive commercial hot embossing facilities. Meanwhile, the process can produce micro-array channels with large area, and the micro-embossing tools can be used repeatedly. Therefore, UFG LZ91 Mg-Li alloy has potential applications in the fabrication of MEMS components with a low cost via the micro-embossing process.

## 5. Conclusions

Micro-array channels on the UFG LZ91 Mg-Li alloy produced by HPT were fabricated by micro-embossing processes with width ranging from 50 to 200 μm using metal dies for. The conclusions are drawn as follows:

(1) Micro-embossing processes are performed on the UFG LZ91 Mg-Li alloy processed by HPT by metal die, and the micro-array channels with widths ranging from 50 μm to 200 μm are fabricated under an applied force of 2 kN with a temperature of 423 K followed by a dwell time of 60 s. The results indicate that embossed micro-channels with all widths were clearly embossed well, with an excellent geometrical transferability without obvious disfigurement, and filled completely.
(2) Micro-embossing of the CG LZ91 Mg-Li alloy produces micro-array channels that are 100 μm in width and are uneven with wrinkles, but the micro-embossing of the UFG LZ91 Mg-Li alloy at the same temperature produces smooth micro-channels. The patterns on the metal mold are fully transferred to the UFG LZ91 Mg-Li alloy plate.
(3) Micro-embossing of UFG LZ91 Mg-Li alloy has good potential applications in mass fabrication of MEMS components. In the future, UFG materials with much finer grains will be used to fabricate much smaller channels.

## Figures and Tables

**Table 1:** Filling quality measurement of micro-array channels after micro-embossing using UFG LZ91 Mg-Li alloy (μm).

Channel Width *w*	Channel Depth *ho*	Filling Height *h*
50	50	~50
100	100	~100
150	150	~150
200	200	~200
